# Out Of Reach: Inequities In The Use Of High-Quality Home Health Agencies

**DOI:** 10.1377/hlthaff.2021.01408

**Published:** 2022-02

**Authors:** Shekinah A. Fashaw-Walters, Momotazur Rahman, Gilbert Gee, Vincent Mor, Michael White, Kali S. Thomas

**Affiliations:** University of Minnesota, Minneapolis, Minnesota.; Brown University, Providence, Rhode Island.; University of California Los Angeles, Los Angeles, California.; Brown University and Providence Veterans Affairs Medical Center, Providence, Rhode Island.; Brown University.; Brown University and Providence Veterans Affairs Medical Center.

## Abstract

Patients receiving home health services from high-quality home health agencies often experience fewer adverse outcomes (for example, hospitalizations) than patients receiving services from low-quality agencies. Using administrative data from 2016 and regression analysis, we examined individual- and neighborhood-level racial, ethnic, and socioeconomic factors associated with the use of high-quality home health agencies. We found that Black and Hispanic home health patients had a 2.2-percentage-point and a 2.5-percentage-point lower adjusted probability of high-quality agency use, respectively, compared with their White counterparts within the same neighborhoods. Low-income patients had a 1.2-percentage-point lower adjusted probability of high-quality agency use compared with their higher-income counterparts, whereas home health patients residing in neighborhoods with higher proportions of marginalized residents had a lower adjusted probability of high-quality agency use. Some 40–77 percent of the disparities in high-quality agency use were attributable to neighborhood-level factors. Ameliorating these inequities will require policies that dismantle structural and institutional barriers related to residential segregation.

Home health is a large and growing segment of the Medicare program.^1^ In 2017 approximately 3.4 million homebound Medicare beneficiaries used home health, costing Medicare $18 billion that year.^1^ Medicare home health provides intermittent skilled nursing care; physical, occupational, and speech therapies; medical social services; and intermittent home health aide services. Home health use and spending are expected to grow as the size of the older adult population increases and the shift toward community-based (versus nursing home) care continues.^2^ Prior research documents considerable geographic variation in the use of home health associated with county sociodemographics and home health agency characteristics.^3,4^ However, geographic differences in home health access and quality are not well understood. Furthermore, home health is a unique health care service in that the care is delivered in patients’ homes rather than in a centralized, physical location such as doctor’s office, hospital, or nursing home. Therefore, much remains to be learned about the association between the local neighborhood and potential disparities in access to high-quality home health agencies by race, ethnicity, and income.^5^

It is important to understand access to high-quality home health care, given the documented associations between poor patient outcomes and agency quality.^6–11^ Prior research suggests that patients receiving home health services from high-rated agencies experience fewer adverse outcomes (for example, hospitalizations, emergency department use, and poorer functional improvement) compared with those receiving services from low-quality agencies.^6–11^ Therefore, an inability to gain access to high-quality home health agencies not only is detrimental to patients but also is costly to the US health care system.

Disparities in access to high-quality care have been documented in other long-term care settings (for example, nursing homes);^12,13^ however, there is limited evidence on disparities in access to home health. Research focused on nursing homes suggests that racial and ethnic minority and lower-income older adults are more likely to receive care in low-quality facilities than White and higher-income older adults.^12^ Research also suggests that home health agencies serving higher proportions of Black and low-income older adults are lower quality than those with lower proportions of such patients.^6^ It is unclear why this higher concentration of vulnerable beneficiaries in lower-quality home health agencies exists, although the literature suggests that this could be a result of vulnerable populations having decreased access to high-quality home health agencies.^6^ More generally, there is agreement that the concentration of racial and ethnic minority groups into segregated neighborhoods is largely due to a lasting legacy of structural racism that concentrates exposures to stressors and poorer services and diminished neighborhood resources.^5,14^

Previous research reveals that neighborhoods with a greater share of Black, Hispanic, and low-income residents have poor access to high-quality hospitals, primary care physicians, nursing homes, and community-based long-term services and supports (for example, assisted living).^5,13,15–19^ Furthermore, during the past two decades, Black and Hispanic older adults have been entering and residing in nursing homes at higher rates than their White counterparts,^13,20^ leading researchers to speculate that this trend may be a result of inequitable access to community-based long-term services and supports, such as home health.^21^

However, there are no studies, to our knowledge, that examine differences in access to high-quality home health agencies and how these differences vary by individual- and neighborhood-level characteristics (for example, racial composition) while accounting for differences in patients’ health status and sociodemographics. Understanding the mechanisms for disparities in access to high-quality home health agencies within (the individual level) and between neighborhoods can guide policies designed to decrease such disparities and achieve equitable health outcomes.

We examined the associations between Medicare home health patients’ individual- and neighborhood-level characteristics and receipt of care from high-quality home health agencies, as defined by their publicly reported Quality of Patient Care star ratings. On the basis of prior literature, we hypothesized that within and between neighborhoods, marginalized home health patients (that is, patients who are Black, Hispanic, or low income) would be less likely to receive care from high-quality home health agencies than their more advantaged counterparts (that is, patients who are White or higher income). We also hypothesized that patients residing in neighborhoods with more Black, Hispanic, or socioeconomically disadvantaged residents would be less likely to receive care from high-quality home health agencies than beneficiaries in less diverse and more advantaged neighborhoods. Last, we hypothesized that much of the observed individual-level disparities would be attributable to neighborhood-level factors because we expect that fewer high-quality home health agencies serve lower-resourced areas with higher concentrations of marginalized populations.

## Study Data And Methods

National administrative data came from the 2016 Medicare Beneficiary Summary File, the 2016 Outcome and Assessment Information Set (OASIS), the 2016–18 Centers for Medicare and Medicaid Services (CMS) Care Compare website, the 2015 ZIP Code Tabulation Area (ZCTA) Social Deprivation Index (SDI),^22^ and the 2015 American Community Survey (ACS) five-year estimates. We linked the OASIS and Medicare Beneficiary Summary File data using beneficiary identifiers and then used CMS provider numbers to link the OASIS and CMS Care Compare data. Finally, we used the 2015 Uniform Data Set mapper ZCTA crosswalk to link the patients’ ZIP codes found in the Medicare Beneficiary Summary File with the ACS and SDI data.

Medicare-certified home health agencies are required to submit OASIS assessments for all Medicare beneficiaries receiving skilled home health services. We used OASIS data to identify individual home health recipients, the agency serving them, and other individual-level information (for example, race, health status, and living arrangements). Since 2015 all Medicare-certified home health agencies have a publicly reported star rating, which is updated quarterly on the CMS Care Compare website. Details on the Quality of Patient Care star ratings, which range from 1 (poor quality) to 5 (excellent quality), are available on the CMS website.^23^

The outcome variable was receipt of care from a high-quality home health agency—a dichotomous variable that identified agencies as high quality if their average Quality of Patient Care star rating was greater than 3.5 stars across twelve quarters of data (January 2016–December 2018); otherwise, agencies were identified as not high quality. CMS recognizes home health agencies of “above average” quality as having ratings of greater than three stars.^24^

The independent variables were measured at the beneficiary level and described beneficiaries’ race, ethnicity, socioeconomic status, and neighborhood characteristics. To identify non-Hispanic Black, Hispanic, and non-Hispanic White beneficiaries, we used self-reported race and ethnicity from OASIS.^25^ Throughout the article, “Black” and “White” refer to non-Hispanic beneficiaries unless otherwise specified. A beneficiary’s low-income status was determined by dual enrollment in Medicare and Medicaid, participation in the Medicare Part D low-income cost-sharing subsidy, or both at the time of home health initiation, which allowed for more uniform and potentially sensitive measurement of low-income status.

We included two neighborhood (defined by ZCTA) characteristics: racial composition and socioeconomic disadvantage. We used the ACS data to operationalize racial composition as the proportion of Black and Hispanic residents in the neighborhood. Socioeconomic disadvantage was operationalized using two variables: the SDI score and the percentage of residents with incomes below 100 percent of the federal poverty level.^22^ The SDI score, created via a series of items, is a composite centile that divides the ordered set of sociodemographic measures into 100 parts, making the score easily interpretable by way of an underlying scale. The higher the SDI score, the more socioeconomically disadvantaged the neighborhood.

The following covariates were included: sex, age, Medicare Advantage enrollment, living alone, having caregiver support, need for assistance in activities of daily living, cognitive impairment, previous discharge location, risk for hospitalization, health status risk factors (for example, obesity and smoking), and presence of a surgical wound.

### STUDY SAMPLE

Our sample consisted of 3,111,537 Medicare-enrolled home health patients ages sixty-five and older with a start-of-care assessment in 2016. We included only the first such assessment per person in 2016. We excluded home health patients residing in congregate housing such as assisted living (*n* = 523,852) to focus on community dwellers in “noninstitutional” settings who had more control over the home health agency used. Because of sample limitations, we also excluded patients who were Asian, American Indian, Pacific Islander, and other race. For more stable results in our neighborhood-level analysis, we excluded ZCTAs with fewer than fifty home health patients (*n* = 17,226); our final sample at the neighborhood level was 13,750 neighborhoods.

### ANALYSIS

We conducted three main analyses. First, we estimated the relationship between individual characteristics (including race or ethnicity) and receiving care from a high-quality home health agency, using a covariate-adjusted linear probability regression model with ZCTA fixed effects.

Second, we estimated and compared models with and without the ZCTA fixed effects. The fixed effects account for both observed and unobserved neighborhood characteristics. Comparing the estimates across the two models allowed us to quantify the explanatory power of neighborhoods on the observed disparities in use of high-quality home health agencies.

Finally, at the neighborhood level we examined the relationship between receiving care from a high-quality home health agency in a neighborhood and neighborhood characteristics (including racial and ethnic composition). We first predicted the receipt of care from a high-quality home health agency based on beneficiary characteristics (including race or ethnicity) and determined the average predicted probability of using a high-quality agency for residents within each neighborhood. We then calculated the adjusted probability of high-quality agency use for each neighborhood by dividing the observed rate of use by the average predicted probability of use, multiplied by the national rate of use. All analyses were conducted using Stata, version 16. See the online appendix for more detail on the methods, including data linkages, variables, and models.^26^

### LIMITATIONS

This observational study relied on association-based analyses; therefore, we were unable to draw causal conclusions. Furthermore, although the star measures were “risk adjusted” in an attempt to control for differences in patient populations across home health agencies, our analyses could not untangle whether disadvantaged patients were more likely to gain access to low-quality home health agencies and whether agencies that serve disadvantaged patients were more likely to receive low quality scores. Importantly, the star ratings we used are only one measure of quality; future studies should consider examining other measures of quality, such as the patient satisfaction star ratings available on CMS Care Compare. In addition, although ZCTAs are not an ideal unit of geography to identify neighborhoods, they have been used in other studies^27^ and were the best available units because of data constraints. Furthermore, we excluded Asian Americans, Pacific Islanders, and Native Americans, who make up less than 3 percent of home health patients in our data; future work is needed to understand home health use among these populations. Finally, this work using 2016 data predated the 2020 home health Patient-Driven Groupings Model,^28^ which may influence patterns of use as a result of payment changes, as well as the 2022 national rollout of the Home Health Value-Based Purchasing model, which may affect home health agency quality performance and star ratings.^29^ The Patient-Driven Groupings Model is a thirty-day case-mix-adjusted payment model that relies mostly on clinical and patient characteristics, and the Home Health Value-Based Purchasing model gives home health agencies incentives to provide higher-quality and more efficient care. Future work to understand the impact of these initiatives on disparities in access is warranted.

## Study Results

Of the 3,111,537 Medicare beneficiaries included in our sample with a home health start-of-care assessment in 2016, 81 percent were White, 12 percent were Black, 7 percent were Hispanic, and 26 percent were low income (exhibit 1). Overall, 46 percent of the sample received services from high-quality home health agencies: 48 percent of White, 42 percent of Black, 36 percent of Hispanic, and 43 percent of low-income beneficiaries used high-quality home health agencies. Black and Hispanic home health patients lived in neighborhoods with a greater average number of home health agencies per 1,000 older adults, but a smaller percentage of those were high-quality home health agencies. Other covariates are also displayed by race, ethnicity, and socioeconomic position in exhibit 1.

Within neighborhoods with the same Social Deprivation Index score and proportions of Black residents, Hispanic residents, or residents living below 100 percent of poverty, there was a visible individual-level racial, ethnic, and socioeconomic difference: Black, Hispanic, and low-income home health patients had lower use of high-quality home health agencies, on average, than did their White and higher-income counterparts (exhibit 2). More detailed findings are in supplemental exhibit 2.^26^ Within neighborhoods, having an increased share of Black, Hispanic, and lower-income residents was associated with decreased use of high-quality home health agencies for all home health patients regardless of patients’ race, ethnicity, and low-income status.

After individual-level characteristics were controlled for, Black, Hispanic, and low-income home health patients were significantly less likely to receive care from high-quality home health agencies compared with their White and higher-income counterparts (exhibit 3). The unadjusted difference between Black and White home health patients was 5.5 percentage points. In addition, there was a 11.9-percentage-point difference between Hispanic and White home health patients and a 3.9-percentage-point difference between higher-income and low-income patients in their use of high-quality home health agencies.

After individual characteristics were adjusted for, Black home health patients had a 5.6-percentage-point lower probability of high-quality home health agency use, Hispanic patients had a 10.9-percentage-point lower probability, and low-income patients had a 2.0-percentage-point lower probability compared with their counterparts. After neighborhood fixed effects were included, Black home health patients had a 2.2-percentage-point lower probability of high-quality home health agency use, Hispanic patients had a 2.5-percentage-point lower probability, and low-income patients had a 1.2-percentage-point lower probability as compared with their counterparts. All differences were statistically significant (*p* < 0.001). Adjustment for neighborhood characteristics with neighborhood fixed effects conveyed that 61 percent of the Black-White disparity, 77 percent of the Hispanic-White disparity, and 40 percent of the socioeconomic disparity was related to neighborhood factors.

Using the risk-adjusted probability of high-quality home health agency use, exhibit 4 demonstrates that an increase in the proportion of Black residents, Hispanic residents, and neighborhood socioeconomic disadvantage (as measured by the SDI score and the proportion of residents with incomes below 100 percent of poverty) was associated with a decrease in the probability of use of high-quality home health agencies.

## Discussion

In this study we found that Black, Hispanic, and low-income Medicare home health patients, along with patients residing in neighborhoods with a higher share of Black, Hispanic, or socioeconomically disadvantaged (for example, lower-income) residents were less likely to receive care from high-quality home health agencies. These findings are consistent with prior literature.^6^

However, we have provided new evidence in the following three ways. First, differences in access exist even after patients’ health status and care needs are adjusted for, thus indicating a true racial, ethnic, and socioeconomic disparity in access to higher-quality home health agencies. Second, even when Black, Hispanic, and lower-income home health patients reside in neighborhoods with a greater number of high-quality home health agencies, disparities exist in access to these agencies, thus illustrating that differential geographic access is not the only cause of decreased access. Finally, disparities exist between neighborhoods. Between 40 percent and 77 percent of the observed individual-level racial, ethnic, and socioeconomic disparities in the use of high-quality home health agencies was attributable to neighborhood-level factors. This suggests that racial, ethnic, and socioeconomic inequities in access to high-quality home health care are robust and pervasive, and they put high-quality home health agency services “out of reach” for the most vulnerable Medicare home health patients as characterized by race, ethnicity, and income status.

When one is considering the possible mechanisms behind inequities in access to high-quality home health agencies observed in this study, it is difficult to separate patient- and provider-driven factors. It may be the case that the observed disparities reflect Black, Hispanic, and lower-income home health patients’ choice of agencies or their lower use and limited understanding of publicly reported quality information compared with their White and higher-income counterparts.^30–32^ More research is needed to understand these mechanisms within home health. Regardless, patient choice is unlikely to explain all of the inequities revealed in this study, particularly given the finding that a meaningful amount of the disparities observed (40–77 percent) is attributable to the neighborhoods in which home health patients reside. Therefore, providers’ roles in home health access inequities must also be considered.

The observed disparities in access to high-quality home health agencies may be manifestations of institutionalized racism and discrimination. *Institutionalized racism* is defined as a system of structures, policies, practices, and norms that assign value and provide opportunity based on race.^33^ As has been suggested in prior work, it may be the case that the observed disparity is related to providers’ preferences to provide access to socioeconomically advantaged patients and patients living in neighborhoods with higher socioeconomic status. Such patients are perceived to have the characteristics that are the least likely to lower the provider’s quality ratings; caring for such patients may be more lucrative when pay-for-performance initiatives are initiated.^30,31,34–39^

Research also suggests that patients’ race, ethnicity, and socioeconomic status affect providers’ perceptions of their risk behaviors and potential adherence to medical advice.^37^ The within-neighborhood racial and ethnic disparities observed in our study, although somewhat narrow, may also reflect the effects of interpersonal racism and discrimination by home health agencies and nurses against racial and ethnic minority home health patients.^40^ In health equity research, it is important when examining differences across racial groups and socioeconomic status not to focus on the magnitude of the estimate but rather to center on the margins, as any difference that is potentially driven by injustices and rooted in racism is of great concern.^41^

Although there has been limited work examining the impact of residential segregation on access to home health agencies, previous research suggests that agencies left socioeconomically disadvantaged markets after the initiation of home health public reporting in 2003 in an effort to protect their reputation and quality ratings.^35^ From what is known about the linkage between residential segregation and socioeconomic disadvantage, these socioeconomically disadvantaged markets may have been racially segregated as well.^5^ Further, qualitative research findings suggest that disparities in access may exist because home health agency staff (for example, nurses and aides) decline to provide services in predominantly Black, Hispanic, and lower-income neighborhoods because of their perceptions of safety concerns and hazardous conditions.^40,42^

Our finding that unmeasured and measured neighborhood factors play a large role in the observed disparity within neighborhoods aligns with the findings of prior research showing that health care providers are less likely to serve predominantly Black, Hispanic, or disadvantaged neighborhoods.^5,13,15–18,43^ The resulting lack of access to services may further exemplify the impact of institutional racism as a fundamental cause of disparities^5^—in this case, disparities in the use of high-quality home health agencies. These findings suggest the need to further examine how access to high-quality agencies is shaped by the neighborhoods where patients live.

To address the disparities observed in our study, policy makers should consider incentivizing the delivery of high-quality home health services in predominantly Black, Hispanic, and low-income neighborhoods. This could be achieved through a program similar to Medicaid Disproportionate Share Hospital payments, which intend to improve access to care for lower-income and uninsured patients. In addition, CMS could consider reimbursing home health agencies for the use of clinician escorts or security personnel for nurses serving in “high-risk” areas with concerns about violence.^40,42,44^ Improving home health access, especially for the most vulnerable, is increasingly important considering COVID-19 and the increased demand for home health services in lieu of nursing home care,^45^ as well as the Biden administration’s Build Back Better Act, which dedicates $150 billion to transform and bolster the home-based care industry.^46^ The resources provided through Build Back Better offer the opportunity to reduce waiting lists for home care services and improve pay for home care professionals. Federal administrators should carefully consider how these funds can address inequities in access and enable all older adults to receive high-quality home health services.

Our findings may also have implications for market-based reforms such as the Home Health Value-Based Purchasing initiative^47^ and Home Health Compare five-star ratings. These programs are “colorblind” market-based reforms intended to reward home health agencies for high-quality care while penalizing providers with lower quality. However, similar programs have been shown to exacerbate health disparities in home health and other settings.^30,48,49^ For example, after the introduction of five-star ratings in nursing homes, high-quality facilities selectively admitted more profitable residents while avoiding Medicaid residents,^50^ exacerbating existing socioeconomic disparities in the use of high-quality facilities.^30^

Understanding disparities in access to care and how these disparities relate to market-based reforms is an important next step in home health research. Improved understanding will allow policy makers to consider strategies to mitigate disparities in access, quality, and outcomes while supporting the neighborhoods, historically underserved patient populations, and providers who need the most support to reduce and possibly eliminate disparities.^7^

## Conclusion

This study serves as a call to action for policy makers and the Medicare home health program to urgently consider reducing racial and socioeconomic disparities in access to high-quality home health care. Mitigating these disparities will require policies that dismantle structural and institutional barriers of racism, incentivize serving the underserved, and reallocate resources to the most vulnerable areas and patient populations. Ensuring equitable access and aging for all older adults means taking the necessary steps to put high-quality home health agencies within the reach of the most marginalized.

## Supplementary Material

Supplement and Appendix

## Figures and Tables

**EXHIBIT 2:**
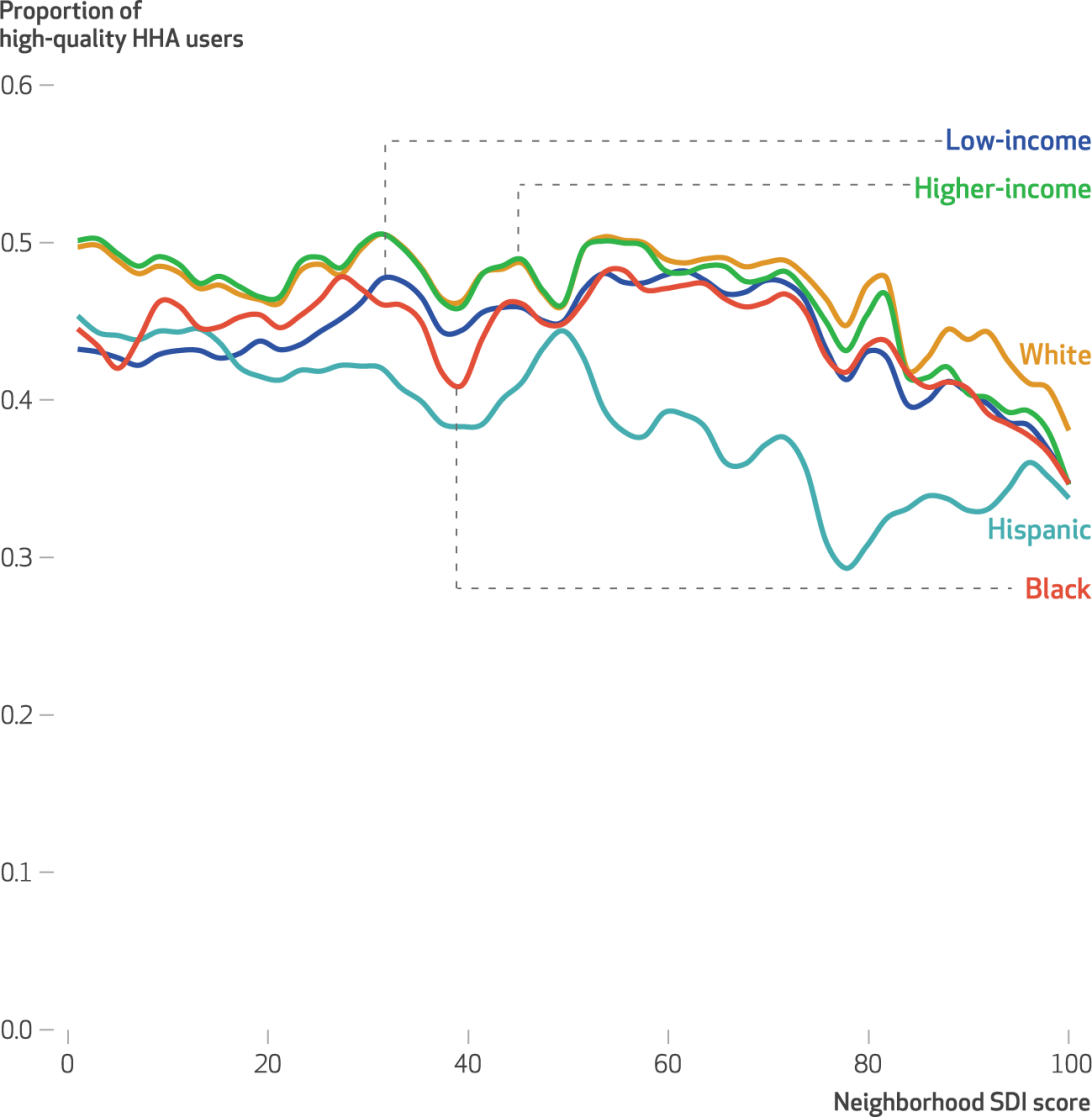
Unadjusted relationships between neighborhood social deprivation and high-quality home health agency (HHA) use, by Medicare beneficiary race, ethnicity, and socioeconomic position **source** Authors’ analysis of data from the 2016 Medicare Beneficiary Summary File, the 2016 Outcome and Assessment Information Set (OASIS), the 2016–18 Centers for Medicare and Medicaid Services (CMS) Care Compare, the 2015 ZIP Code Tabulation Area (ZCTA) Social Deprivation Index (SDI), and the 2015 American Community Survey five-year estimates. **notes** High-quality HHAs have CMS Quality of Patient Care star ratings greater than 3.5 stars. Race and ethnicity are self-reported through OASIS. “Low-income” participants identified a beneficiary as having dual enrollment in Medicare and Medicaid, participation in the Medicare Part D low-income cost-sharing subsidy, or both; “higher-income” beneficiaries are all others. Neighborhoods are defined by ZCTA. The SDI is a centile score ranging from 1, the least socially deprived, to 100, the most socially deprived.

**EXHIBIT 4:**
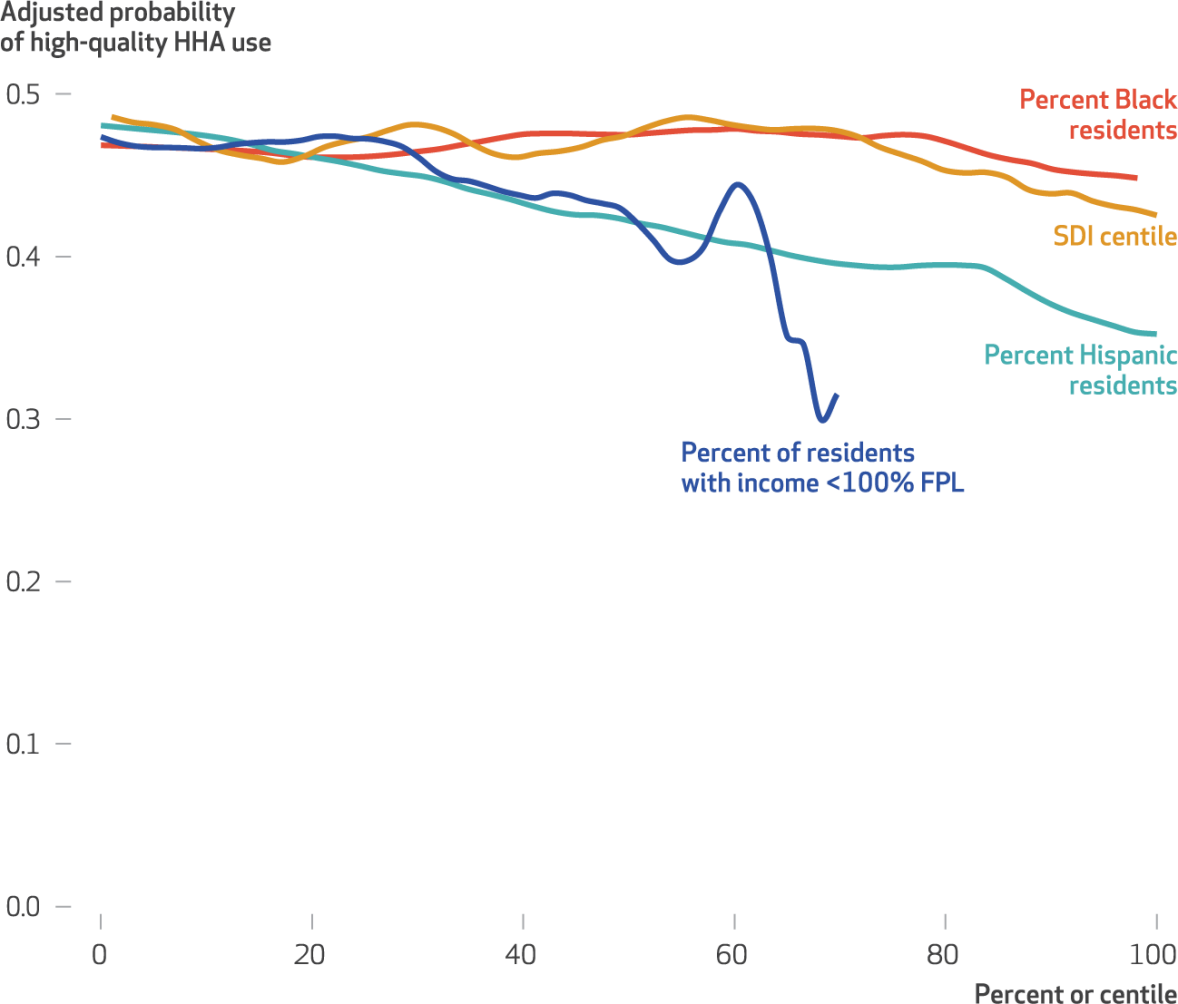
Relationships between neighborhood-level risk-adjusted probability of high-quality home health agency (HHA) use and neighborhood characteristics, by Medicare beneficiary race, ethnicity, and socioeconomic position **source** Authors’ analysis of data from the 2016 Medicare Beneficiary Summary File, the 2016 Outcome and Assessment Information Set (OASIS), the 2016–18 Centers for Medicare and Medicaid Services (CMS) Care Compare, the 2015 ZIP Code Tabulation Area (ZCTA) Social Deprivation Index (SDI), and the 2015 American Community Survey five-year estimates. **notes** High-quality HHAs have CMS Quality of Patient Care star ratings greater than 3.5 stars. Race and ethnicity are self-reported through OASIS. “Low-income” participants identified a beneficiary as having dual enrollment in Medicare and Medicaid, participation in the Medicare Part D low-income cost-sharing subsidy, or both; “higher-income” beneficiaries are all others. Neighborhoods are defined by ZCTA. The SDI is a centile score ranging from 1, the least socially deprived, to 100, the most socially deprived. FPL is federal poverty level.

**EXHIBIT 1:** Medicare home health patient and neighborhood characteristics, by race, ethnicity and socioeconomic position, 2016

	Home health patients					
Characteristics	White	Black	Hispanic	Higher-income	Low-income	All
No. of patients	2,535,520	365,179	210,838	2,298,171	813,366	3,111,537
Percent of patients	81.5	11.7	6.8	74.0	26.0	—^a^
High-quality HHA patients (%)	47.8	42.3	35.9	47.3	43.4	46.3
Female (%)	60.4	65.4	62.0	57.8	70.4	61.1
Average age (years)	79.5	77.6	78.5	79.7	77.9	79.2
Race (%)						
White	—^a^	—^a^	—^a^	89.2	59.8	81.5
Black	—^a^	—^a^	—^a^	7.8	22.8	11.7
Hispanic	—^a^	—^a^	—^a^	3.0	17.5	6.8
Low-income(%)	19.2	50.7	67.3	—^a^	—^a^	26.1
In Medicare Advantage (%)	26.2	33.8	34.0	26.5	30.7	27.6
Lives alone (%)	29.4	27.5	19.5	26.5	34.1	28.5
Has caregiver support (%)	87.1	85.7	88.6	87.5	85.8	87.1
Average ADL score	3.3	3.5	3.4	3.3	3.4	3.4
Cognitively impaired (%)	44.9	50.5	54.3	43.4	53.9	46.2
Discharged from (%):						
Long-term care nursing facility	0.8	0.8	0.6	0.7	1.0	0.8
Skilled nursing facility	18.3	13.8	10.2	17.9	15.2	17.2
Acute hospitalization	49.4	43.5	40.5	50.9	39.9	48.1
Long-term care hospital	0.5	0.7	0.8	0.5	0.6	0.5
Inpatient rehabilitation facility	7.3	6.6	5.5	7.6	5.7	7.1
Psychiatric hospital	0.2	0.2	0.1	0.2	0.2	0.2
At risk for hospitalization (%)	97.6	97.4	97.2	97.4	98.1	97.6
Has health status risk factor (%)	37.0	39.4	28.8	34.8	42.1	36.8
Surgical wound present (%)	30.4	21.1	21.3	32.5	17.6	28.6
Neighborhood social disadvantage						
Average SDI score	44.0	72.7	72.8	44.6	62.8	49.3
Average percent of residents <100% FPL	13.8	23.7	21.9	14.0	19.7	15.5
Neighborhood racial composition, average (%)						
Black residents	8.7	43.6	9.0	11.2	17.3	12.8
Hispanic residents	11.2	13.8	55.1	12.3	20.7	14.5
White residents	73.3	36.6	28.7	69.7	55.4	65.9
Neighborhood HHA composition per 1,000 older adults						
No. of HHAs, average	10.8	15.8	17.0	10.9	14.2	11.8
No. of high-quality HHAs, average	4.7	5.7	6.0	4.7	5.8	4.9
No. of average-quality HHAs, average	4.8	7.0	6.9	4.9	6.1	5.2
No. of low-quality HHAs, average	1.1	2.7	3.9	1.3	2.1	1.5
No. of HHAs with no quality rating, average	0.1	0.3	0.3	0.1	0.2	0.1

**source** Authors’ analysis of data from the 2016 Medicare Beneficiary Summary File, the 2016 Outcome and Assessment Information Set (OASIS), the 2016–18 CMS Care Compare, the 2015 ZIP Code Tabulation Area (ZCTA) Social Deprivation Index (SDI), and the 2015 American Community Survey five-year estimates. **notes** *N* = 3,111,537. Race and ethnicity are self-reported through OASIS. “Low-income” participants identified a beneficiary as having dual enrollment in Medicare and Medicaid, participation in the Medicare Part D low-income cost-sharing subsidy, or both; “higher-income” beneficiaries are all others. Neighborhoods are defined by ZCTA. The SDI is a centile score ranging from 1, the least socially deprived, to 100, the most socially deprived. High-quality home health agencies (HHAs) have CMS Quality of Patient Care star ratings greater than 3.5 stars. See the appendix for more detail on the variables (note 26 in text). ADL is activities of daily living. FPL is federal poverty level.

a Not applicable.

**EXHIBIT 3:** Probability of high-quality home health agency (HHA) use for individual Medicare home health patients by race, ethnicity, and socioeconomic position, comparing the inclusion of neighborhood characteristics, 2016

Characteristics	Black	White	Diff.	Hispanic	White	Diff.	Low-income	Higher income	Diff.
Beneficiaries using high-quality HHAs (unadj.)	42.26%	47.75%	−5.5	35.86%	47.75%	−11.9	43.45%	47.31%	−3.9
Beneficiaries using high-quality HHAs without neighborhood fixed effects (adj.)	42.08%	47.70%	−5.6****	36.79%	47.70%	−10.9****	44.80%	46.83%	−2.0****
Beneficiaries using high-quality HHAs with neighborhood fixed effects (adj.)	44.53%	46.73%	−2.2****	44.22%	46.73%	−2.5****	45.40%	46.62%	−1.2****

**source** Authors’ analysis of data from the 2016 Medicare Beneficiary Summary File, the 2016 Outcome and Assessment Information Set (OASIS), the 2016–18 Centers for Medicare and Medicaid Services (CMS) Care Compare, the 2015 ZIP Code Tabulation Area (ZCTA) Social Deprivation Index, and the 2015 American Community Survey five-year estimates. **notes** *N* = 3,111,537. Unit of analysis was the person level. High-quality HHAs have CMS Quality of Patient Care star ratings greater than 3.5 stars. Control variables are in the Study Data and Methods section. See the appendix for more detail on the variables (note 26 in text). Differences are presented in terms of percentage points except for the row labeled “Disparity due to neighborhood characteristics,” where the differences are relative. The Stata MARGINS command was used to calculate the predicted percentage of high-quality HHA use. Significance tests were conducted on adjusted results.

**** *p* < 0.001
