# Supplementary material for: Out Of Reach: Inequities In The Use Of High-Quality Home Health Agencies
Source: Health Aff (Millwood). Author manuscript; Available in PMC 2022 Feb 28. (PMC8883595; doi:10.1377/hlthaff.2021.01408)
Supplement: Supplement and Appendix [file NIHMS1778634-supplement-Supplement_and_Appendix.pdf]

Appendix

1. Supplemental Methods
2. Supplemental Exhibit 2: Unadjusted relationships between neighborhood factors and high-quality home health agency use, by beneficiary race, ethnicity, and socioeconomic position

Supplemental Methods

We used a cross-sectional observational study design to evaluate the probability of receiving care from a high-quality home health agency for Medicare beneficiaries of different races, ethnicities, socioeconomic statuses, and neighborhood characteristics.

Data National administrative data came from the 2016 Medicare Beneficiary Summary File, the 2016 Outcome and Assessment Information Set, the 2016-18 Centers for Medicare and Medicaid Services (CMS) Care Compare website, the 2015 ZIP Code Tabulation Area (ZCTA) Social Deprivation Index, and the 2015 American Community Survey (ACS) five-year estimates.

The Medicare Beneficiary Summary File contains beneficiaries' demographic characteristics, enrollment information, and ZIP codes. Medicare-certified home health agencies are required to submit Outcome and Assessment Information Set assessments for all Medicare beneficiaries receiving skilled home health services. We used the Outcome and Assessment Information Set to identify individual home health recipients, the home health agency serving them, as well as other beneficiary-level information (e.g., health status, living arrangements). These data were linked to the Medicare Beneficiary Summary File using the beneficiary ID number.

Since 2015, all Medicare-certified home health agencies have a publicly reported star rating, which is updated quarterly on the CMS Care Compare website (https://www.medicare.gov/care-compare). The Home health agencies' star ratings were linked to the beneficiary data using the Medicare provider number for each home health agency. The quality-of-patient-care star ratings, which range from 1-5 with half-star intervals, were used to characterize home health agency quality, and were calculated by CMS using various process

and outcome measures from the Outcome and Assessment Information Set and Medicare claims, details are available on CMS' website. The quality-of-care patient stars include 9 measures of quality: (1) timely initiation of care; (2) drug education on all medications provided to patient/caregiver; (3) influenza immunization received for current flu season; (4) improvement in ambulation; (5) improvement in bed transferring; (6) improvement in bathing; (7) improvement in pain interfering with activity; (8) improvement in shortness of breath; and (9) acute care hospitalizations.

Last, we used the publicly available Social Deprivation Index data, which uses ACS 5-year estimates to collate information on neighborhood-level poverty status. Neighborhood racial composition is collated from the ACS 5-year estimates available through data.census.gov. The neighborhood details are described in greater detail below. These data were linked to the beneficiary-level data using the ZCTAs.

The outcome variable of interest was receipt of care from a high-quality home health agency - a dichotomous variable that identified home health agencies as high-quality if their average quality of care star rating was greater than 3.5 stars across 12 quarters of data (January 2016 - December 2018); otherwise, home health agencies were identified as non-high-quality. Home health agencies with an average of 3.5 stars were chosen as high-quality home health agencies because CMS recognizes above average quality as having greater than 3 stars.

The independent variables of interest were measured at the beneficiary level and described their race, ethnicity, socioeconomic status, and neighborhood characteristics. To identify non-Hispanic Black, Hispanic, and non-Hispanic White beneficiaries, we used the "gold-standard" self-reported race and ethnicity in the Outcome and Assessment Information Set.

During the start of care home health visit a registered nurse or licensed therapist administers the Outcome and Assessment Information Set assessment and race/ethnicity is obtained by self-report. The Outcome and Assessment Information Set item instructs respondents to “mark all that apply.” Researchers have found that the majority of respondents respond with only a single race or ethnicity. For the purposes of this paper, we recode this variable to be non-Hispanic White, non-Hispanic Black, and Hispanic. We exclude respondents who identify as Asian, Native American/American Indian, or Native Hawaiian as they only comprise 2.85% of the 2016 sample. A beneficiary’s low-income status is determined by dual enrollment in Medicare and Medicaid and participation in the Medicare Part-D low-income cost-sharing subsidy (LIS) at the time of home health initiation. We use the Part-D LIS to capture more potentially low-income patients, as the LIS has a more generous eligibility than Medicaid, does not vary by state, and therefore allows for more uniform and potentially sensitive measure of low-income status.

Neighborhoods were defined by the ZCTAs, which were mapped onto the beneficiaries’ home ZIP code using the Uniform Data Set mapper (https://udsmapper.org/). We used the beneficiary ZIP code that corresponded with the month of their Outcome and Assessment Information Set assessment. We included two neighborhood characteristics: neighborhood racial composition and socioeconomic disadvantage. We used the American Community Survey data to operationalize racial composition as the proportion of Black and Hispanic residents in the neighborhood. Neighborhood socioeconomic disadvantage was operationalized using two variables: (1) the social deprivation index score/centile and (2) the percent of residents living below 100% of the Federal Poverty Level (FPL). The social deprivation index score is a

composite centile that divides the ordered set of sociodemographic measures into 100 parts, making the social deprivation index score easily interpretable by way of an underlying scale. The social deprivation index score is calculated from a composite of the percent of the population: (1) living below 100% FPL; (2) 25 years of age or more with less than 12 years of education; (3) non-employed; (4) unemployed; (5) living in renter occupied housing; (6) living in crowded housing units; (7) without a car; (8) single-parent households with dependents < 18 years. The higher the social deprivation index score, the more socioeconomically disadvantaged the neighborhood. A social deprivation index score of 100 meant that the neighborhood was the most deprived while a score of 1 made it the least deprived.

Several covariates were also included in the study: sex, age, Medicare Advantage enrollment, living alone, caregiver support, need for assistance in activities of daily living, cognitive impairment, previous discharge location, risk for hospitalization, health status risk factors, and surgical wound presence. We controlled for the sex and age of home health patients with data from the Medicare Beneficiary Summary File. We also included beneficiaries' Medicare Advantage enrollment status, at the time of the home health episode, as defined in the Medicare Beneficiary Summary File to control for sociodemographic, health, and utilization differences that exist between Traditional Medicare and Medicare Advantage enrollees. We measured living alone as a reflection of home health patients' social support using the patient living situation variable in the Outcome and Assessment Information Set (oam1100) as home health patients who live with others have been shown to be less likely to experience adverse events than those living alone. In addition, we controlled for caregiver support in activities of daily living assistance, instrumental activities of daily living assistance, medication

administration, medical procedures, equipment management, supervision and safety, and advocacy (oam2100). The support variable is a dichotomous indicating if caregiver aids with any of the areas listed above.

In terms of health status, we controlled for home health patients' need for assistance with their activities of daily living using 8 measures: grooming, dressing lower body, dressing upper body, bathing, toileting, transferring, ambulation, and eating. To calculate our activities of daily living measure, we used a corrected Likert approach where each individual activity of daily living is divided by the highest possible value for that activity of daily living, allowing all the individual activities of daily living to be on the same scale (0-1). We then summed all of the individual activities of daily living and create a score ranging from 0 to 8, where 0 indicates that the home health patient required no assistance with any of the activities of daily living and 8 indicates some level of assistance needed for all activities of daily living.(26) We also controlled for cognitive impairment using the cognitive functioning variable from the Outcome and Assessment Information Set (oam1700). Home health patients are identified as cognitively impaired if the patient requires prompting, assistance, or are totally dependent on care providers due to disturbances (codes 1-4). In addition, we controlled for where a patient was discharged from prior to starting their home health stay, including nursing facilities, rehabilitation facilities, and hospitals (oam1000). Furthermore, we controlled for the patient's risk of hospitalization due to mental/emotional/behavioral declines, frailty, wight loss, multiple hospitalization or emergency department visits, difficulty with medical instructions, polypharmacy, exhaustion, and other unlisted reasons (oam1032). Finally, we controlled for the presence of a surgical wound (oam1340) and other health risk factors, including smoking,

obesity, alcohol dependency, and drug dependency (oam1036). We used the social support and health status measures to control for the way in which they may impact the choice of home health agencies.

Study Sample Our sample consisted of Black, Hispanic, and White Medicare enrolled (both fee-for-service and Medicare Advantage) home health patients aged 65 years and older who had a start-of-care assessment in 2016. We only included the first start-of-care assessment, per person, in 2016. We excluded home health patients residing in congregate housing (e.g., assisted living n=523,852) to focus on community dwellers in “noninstitutional” settings who had more control over the home health agency used. Because of sample limitations, we also excluded patients who were Asian, American Indian, Pacific Islander, and other races (n=96,165). Our analytic sample consisted of 3,111,537 beneficiaries. In our neighborhood-level analysis we exclude ZCTAs with fewer than 50 home health patients (n=17,226) for more stable results; we include a total of 13,750 neighborhoods.

Analysis In addition to the summary statistics we calculated for beneficiaries, home health agencies, and neighborhood characteristics, we also conducted three main analyses. First, we estimated the relationship between beneficiary characteristics and receiving care from a high-quality home health agency at the beneficiary-level using a linear probability regression model with ZCTA fixed-effects and adjustments for all covariates shown here:

$$(1)\ Y_{iz} = \beta_0 + \beta_1 Race_i + \beta_2 SES_i + \beta_3 PATIENT_i + \delta_z + u_{iz}$$

Where Y_{iz} is a binary outcome of high-quality home health service use for individual *i* residing in ZCTA *z*, *RACE* represents the race and ethnicity of each beneficiary, *SES* indicates the

low-income status of each beneficiary, *PATIENT* is a vector for all the beneficiary-level covariates, and δ_z *are the ZCTA fixed effects.*

Second, to determine disparities attributable to beneficiary characteristics net of the neighborhood effects, we estimated models at the beneficiary level with and without the ZCTA fixed effects (δ_z) adjusting for sociodemographic and health characteristics, see equation 1. The fixed effects account for both observed and unobserved neighborhood and state characteristics. Comparing the estimates across the two models (with [β_{FE}] and without [β_{OLS}] fixed effects – see equation 2 below) allowed us to quantify the explanatory power of neighborhoods on the observed disparities in use of high-quality home health agencies.

$$(2)\ Y = \frac{\beta_{OLS} - \beta_{FE}}{\beta_{OLS}} \times\ 100$$

Finally, we examined the relationship between receiving care from a high-quality home health agency in a neighborhood and neighborhood characteristics. To do this, we first calculated the expected rate. To calculate the expected rate, we initially determine the predicted probability of using a high-quality home health agency, using a logistic regression model that accounts for beneficiary characteristics. We were then able to determine the average predicted probability of using a high-quality home health agency for residents within each neighborhood by aggregating the data to the ZCTA/neighborhood level. The expected rate of high-quality home health agency use at the neighborhood-level was then calculated by dividing the neighborhood predicted probability by the total number of home health patients in the neighborhood. Finally, we calculated the adjusted probability of high-quality home health agency use for each neighborhood using the formula shown below:

$$Adjusted\ \Pr(HQ\ HHA\ Use) = \frac{observed\ rate\ of\ HQ\ HHA\ Use}{expected\ rate\ of\ HQ\ HHA\ Use} \times national\ rate\ of\ HQ\ HHA\ Use$$

We present several plots that illustrate the relationship between neighborhood characteristics and the adjusted probability of receiving care from a high-quality home health agency within the neighborhood. All analyses were conducted using STATA 16 and the Brown University Institutional Review Board reviewed and approved this study.

Limitations This observational study relied on association-based analyses—therefore, we were unable to draw causal conclusions. Furthermore, although the star measures were "risk adjusted" in an attempt to control for differences in patient populations across home health agencies, our analyses could not untangle whether disadvantaged patients are more likely to access low-quality home health agencies and whether home health agencies that serve disadvantaged patients are more likely to receive low quality scores. Importantly, the star ratings we used are only one measure of quality; future studies should consider examining other measures of quality, such as the patient satisfaction star ratings available on Care Compare. In addition, although ZCTAs are not an ideal unit of geography to identify neighborhoods, they have been used in other studies and were the best available units because of data constraints. Furthermore, we excluded Asian Americans, Pacific Islanders, and Native Americans, who make up less than 3 percent of home health patients in our data—future work is needed to understand home health use among these populations. Finally, this work predated the 2020 home health Patient-Driven Groupings Model, which may have influenced patterns of use due to payment changes, as well as the 2022 national roll-out of the Home Health Value-Based Purchasing model, which may affect home health agency quality performance and star

ratings. The Patient-Driven Groupings Model is a thirty-day case-mix adjusted payment model that relies mostly on clinical and patient characteristics, and the Home Health Value-Based Purchasing model incentives home health agencies to provider higher-quality and more efficient care. Future work to understand the impact of these federal initiatives on disparities in access is warranted.

Supplemental Exhibit 2 Unadjusted relationships between neighborhood factors and high-quality home health agency use, by beneficiary race, ethnicity, and socioeconomic position

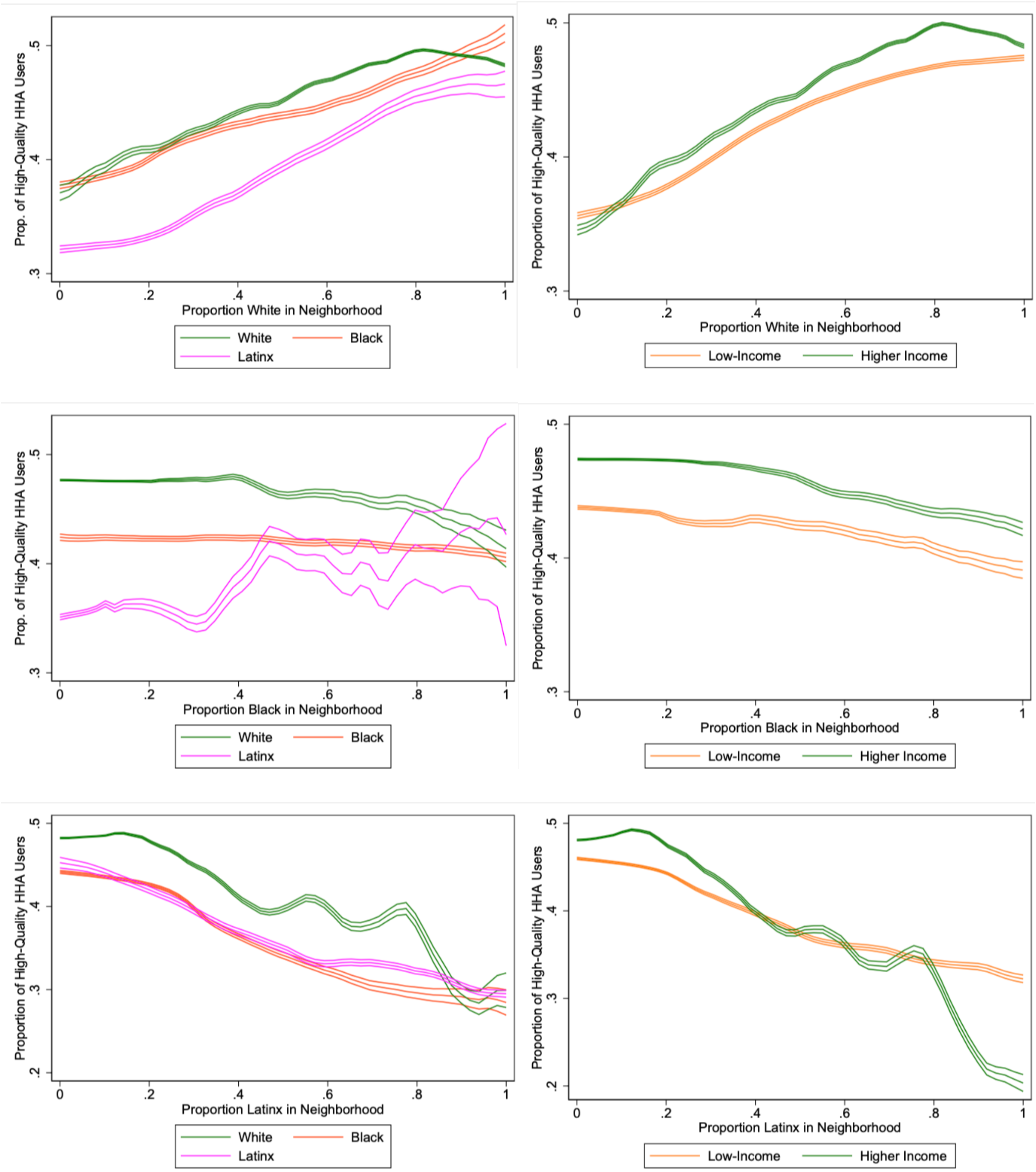

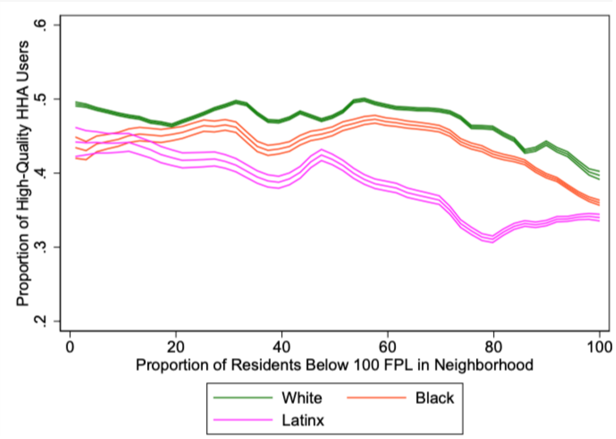

Proportion of High-Quality HHA Users
.2
.3
.4
.5
.6
0
20
40
60
80
100
Proportion of Residents Below 100 FPL in Neighborhood
White
Black
Latinx

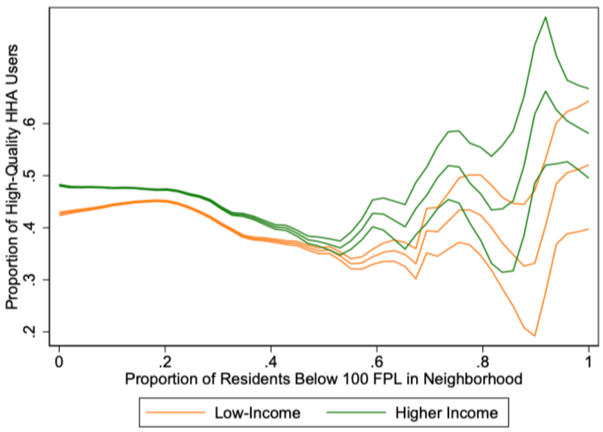

Proportion of High-Quality HHA Users
.2
.3
.4
.5
.6
0
.2
.4
.6
.8
1
Proportion of Residents Below 100 FPL in Neighborhood
Low-Income
Higher Income
